# D3K: The Dissimilarity-Density-Dynamic Radius K-means Clustering Algorithm for scRNA-Seq Data

**DOI:** 10.3389/fgene.2022.912711

**Published:** 2022-07-01

**Authors:** Guoyun Liu, Manzhi Li, Hongtao Wang, Shijun Lin, Junlin Xu, Ruixi Li, Min Tang, Chun Li

**Affiliations:** ^1^ School of Mathematics and Statistics, Hainan Normal University, Haikou, China; ^2^ Key Laboratory of Data Science and Smart Education, Ministry of Education, Hainan Normal University, Haikou, China; ^3^ College of Information Science and Engineering, Hunan University, Changsha, China; ^4^ Geneis Beijing Co., Ltd., Beijing, China; ^5^ School of Life Sciences, Jiangsu University, Zhenjiang, China

**Keywords:** Dissimilarity matrix, density, dynamic radius, ScRNA-seq, K-means

## Abstract

A single-cell sequencing data set has always been a challenge for clustering because of its high dimension and multi-noise points. The traditional K-means algorithm is not suitable for this type of data. Therefore, this study proposes a Dissimilarity-Density-Dynamic Radius-K-means clustering algorithm. The algorithm adds the dynamic radius parameter to the calculation. It flexibly adjusts the active radius according to the data characteristics, which can eliminate the influence of noise points and optimize the clustering results. At the same time, the algorithm calculates the weight through the dissimilarity density of the data set, the average contrast of candidate clusters, and the dissimilarity of candidate clusters. It obtains a set of high-quality initial center points, which solves the randomness of the K-means algorithm in selecting the center points. Finally, compared with similar algorithms, this algorithm shows a better clustering effect on single-cell data. Each clustering index is higher than other single-cell clustering algorithms, which overcomes the shortcomings of the traditional K-means algorithm.

## 1 Introduction

Since the start of genome Project, genome sequencing has been carried out rapidly, and a large amount of genome data has been mined. In order to obtain the information needed by people, bioinformatics emerges as The Times require (Li and Wong, 2019; Liu et al., 2021). It is an interdisciplinary subject composed of life science and computer science, which can dig out the biological significance contained in the chaotic biological data (Sun et al., 2022). Transcriptome is an important research field in bioinformatics, which can study gene function and gene structure from an overall level, and reveal specific biological processes and molecular mechanisms in the process of disease occurrence (Qi et al., 2021; Tang et al., 2020). In order to study the transcriptome, it must be sequenced first, but traditional sequencing techniques ignore the critical differences of individual cells, which will mask the heterogeneous expression between cells and make it difficult to detect subtle potential changes (Huang et al., 2017; Liu et al., 2020). To solve this problem, the single cell RNA sequencing (scrNA-SEQ) technology was developed (Qiao et al., 2017).

scRNA-seq is a powerful method for analyzing gene expression patterns and quickly determining the correct gene expression patterns of thousands of single cells (Potter, 2018). By analyzing scRNA-seq data, we can identify rare cell populations, find subgroup types with different functions, and reveal the regulatory relationship between genes. scRNA-seq can not only show the complexity of single-cell horizontal structure but also improve biomedical research and solve various problems in biology (Yang et al., 2019).

Although the research prospect of scRNA-seq is comprehensive, it also brings new problems and challenges (Kiselev et al., 2019). The scRNA-seq data are high-dimensional and noisy (Xu et al., 2020). Therefore, many clustering methods have been proposed to deal with high-dimensional data structures and noise distribution (Jiang et al., 2018; Zhang et al., 2021; Zhuang et al., 2021). Most of the existing scRNA-seq clustering methods can be divided into unsupervised or semi-supervised clustering (Chen et al., 2016). Zhang et al., (2018) et al. proposed an improved K-means algorithm based on density canopy to find the appropriate center point by calculating the density of the sample data set; Li et al. proposed a new improved algorithm based on T-SNE and density canopy algorithm, called density-canopy-K-means (Li et al., 2019). Compared with similar methods, this clustering algorithm shows stable and efficient clustering performance on single-cell data, thus overcoming the shortcomings of traditional methods; Dong and Zhu, (2020) et al. calculated the dissimilarity parameter between each model by calculating the dissimilarity function between samples and selected the maximum dissimilarity parameter value as the initial clustering center point; Zhu (Zhuang et al., 2021) et al. proposed a new sparse subspace clustering method, which can describe the relationship between cells in a subspace; Ruiqing (Zheng et al., 2019) et al. proposed a method for detecting scRNA-seq cell types based on similarity learning. Wang et al., (2022) propose the scHFC, which is a hybrid fuzzy clustering method optimized by natural computation based on Fuzzy C Mean (FCM) and Gath-Geva (GG) algorithms. The FCM algorithm is optimized by simulated annealing algorithm, and the genetic algorithm is applied to cluster the data to output a membership matrix. Gan et al., (2022). propose a new deep structural clustering method for sc RNA-seq data, named scDSC, which integrates the structural information into deep clustering of single cells. The study by Gan et al., (2022) not only explained the cell typing method behaviors under different experimental settings but also provided a general guideline for the choice of the method according to the scientific goal and dataset properties. Li et al., (2019) Surrogate-Assisted Evolutionary Deep Imputation Model (SEDIM) is proposed to automatically design the architectures of deep neural networks for imputing gene expression levels in scRNA-seq data without any manual tuning. Yu et al., (2022)propose a single-cell model-based deep graph embedding clustering (scTAG) method, which simultaneously learns cell–cell topology representations and identifies cell clusters based on a deep graph convolutional network. Li et al., (2021) propose a multiobjective evolutionary clustering based on adaptive non-negative matrix factorization (MCANMF) for multiobjective single-cell RNA-seq data clustering. Peng et al., (2020) compared 12 single-cell clustering methods and found that most of them improved based on the K-means algorithm.

The K-means algorithm (Macqueen, 1966; Lloyd, 1982) was first proposed by Steinhaus in 1955, Lloyd in 1957, Ball and Hall in 1965, and McQueen in 1967 in different scientific fields. Once the algorithm is put forward, it is widely used in various areas because of its simple principle and easy implementation. At the same time, it is also commonly used in scRNA-seq clustering. However, the K-means algorithm still has some problems. Including that the value of K is difficult to determine, the clustering result depends on the selection of the initial center point, and it is easy to fall into the optimal local solution. In addition, the K-means algorithm is sensitive to noise points and outliers, and it is not practical for nonconvex data sets or data with too significant differences in category size. These problems will have a particular impact on the clustering results. To solve this problem, many workers have carried out a lot of research.

Due to the high-dimensional characteristics of single cells, we reduce the dimension of data sets and then cluster them, which can not only improve the clustering effect but also visually analyze the clustering results. This technology has been widely used in scRNA-seq clustering. Common dimensionality reduction algorithms include Principal Components Analysis (PCA), Locality Preserving Projections (LPP), t-distributed Stochastic Neighbor Embedding (t-SNE), Multidimensional Scaling (MDS), Isometric feature mapping (Isomap), and Locally Linear Embedding (LLE).

Based on dimension reduction, we propose a scRNA-seq clustering method: The dissimilarity-Density-Dynamic Radius-K-means algorithm. The algorithm obtains a set of initial center points by calculating the product of dissimilarity density *ρ*, average dissimilarity of candidate clusters α, and disparity of candidate clusters s. At the same time, the algorithm can optimize the clustering results by adjusting the dynamic radius parameters. We apply this algorithm to single-cell data sets, and the obtained indicators (NMI, FMeasure_node, Accuracy, and RandIndex) are superior to those of other algorithms. They can be used as an effective tool for scRNA-seq clustering.

The main significance of this study lies in the establishment of a clustering model based on single-cell sequencing data, which can be used to cluster cells with similar gene expression patterns into the same cell type so as to infer cell functions and understand the correlation between diseases and genomic characteristics. A more precise and unbiased classification of cells would have a huge impact in oncology, genetics, immunology, and other research fields.

## 2 Materials and Methods

### 2.1 Theoretical Presentation

The K-means algorithm will randomly select K points as initial center points when clustering, which will make the algorithm fall into optimal local solution, and the obtained clustering distribution is not optimal. It is possible to divide a smaller group into one cluster and a large cluster into several small groups. Therefore, the initial center point of the optimal group should meet the following requirements: the difference between the initial center point and other sample points in the group should be as slight as possible; The difference to sample points between the groups is as large as possible.

In thisarticle , the concept of dissimilarity is used when selecting the center point. The so-called dissimilarity is the dissimilarity between two objects, and its expression form is a n×n matrix

$$\left[\begin{array}{cc} \begin{array}{cc} d\left(a_{1},a_{1}\right) & d\left(a_{1},a_{2}\right)\\ d\left(a_{2},a_{1}\right) & d\left(a_{2},a_{2}\right) \end{array} & \begin{array}{cc} \cdots & d\left(a_{1},a_{2}\right)\\ \cdots & d\left(a_{2},a_{n}\right) \end{array}\\ \begin{array}{cc} \vdots & \vdots \\ d\left(a_{n},a_{1}\right) & d\left(a_{n},a_{2}\right) \end{array} & \begin{array}{cc} \ddots & \vdots \\ \cdots & d\left(a_{n},a_{n}\right) \end{array} \end{array}\right],$$

where $d\left(a_{i},a_{j}\right)$ represents the degree of dissimilarity between objects $a_{i}$ and $a_{j}$ , which is usually a non-negative value. The more similar the two things are, the closer the case is to 0; Otherwise, the closer the matter is to 1. We find that if the dissimilarity density ρ of a point is greater, the fact is more likely to become the initial center point.

The dissimilarity density ρ of sample points $x_{i}$ is the number of samples whose dissimilarity with sample objects $x_{i}$ less than the dynamic radius r . Because there are often some noise points in single-cell data sets, if the average dissimilarity is taken as the radius, this fixed-radius algorithm will make the dissimilarity density ρ inaccurate and affect the selection of the initial center point. At the same time, the fixed radius will also cause the number of clusters to be unsatisfactory. Therefore, the traditional fixed-radius method is no longer suitable for single-cell clustering. If it is set to the dynamic radius r , it can effectively solve this problem and is more conducive to single-cell data clustering. The dynamic radius here is the ratio of the average dissimilarity between samples to the dynamic parameters T . The degree of dissimilarity is a model which fully considers the comprehensive distance and dynamic radius, constructed the dissimilarity matrix, and converts the single-cell data into a phase dissimilarity matrix. It can be used to better judge the differences between cells, not just by the distance between them.

### 2.2 Basic Definitions

$X=\left\{x_{1},x_{2},\cdots ,x_{n}\right\}$ is set as the sample data set to be clustered, where $x=\left\{x_{i1},x_{i2},\cdots ,x_{ip}\right\},i\in \left\{1,2,\cdots ,n\right\}$ , and ρ is the number of attributes.

DEFINITION 1 Dissimilarity $d_{ij}$ between sample points $x_{i}$ and $x_{j}$ :

$$d_{\mathrm{ij}}=\sum_{\mathrm{s=1}}^{p}d_{\mathrm{ij}}^{\left(s\right)},$$ (1)

among them

$$d_{\mathrm{ij}}^{\mathrm{(s)}}=\frac{\left|x_{\mathrm{is}}-x_{\mathrm{js}}\right|}{\max\left\{x_{\mathrm{rs}}\right\}\mathrm{-min}\left\{x_{\mathrm{rs}}\right\}}$$ (2)

represents the dissimilarity of the s th attribute between the sample point and, $x_{rs}$ is all the values of the s th attribute.

DEFINITION 2 Constructing dissimilarity matrix d :

$$\mathrm{d=}\left[\begin{array}{cc} \begin{array}{cc} 0 & d_{12}\\ d_{21} & 0 \end{array} & \begin{array}{cc} d_{13} & \begin{array}{cc} \cdots & d_{1n} \end{array}\\ d_{23} & \begin{array}{cc} \cdots & d_{2n} \end{array} \end{array}\\ \begin{array}{cc} d_{31} & d_{32}\\ \begin{array}{c} \vdots \\ d_{n1} \end{array} & \begin{array}{c} \vdots \\ d_{n2} \end{array} \end{array} & \begin{array}{cc} 0 & \begin{array}{cc} \cdots & d_{3n} \end{array}\\ \begin{array}{c} \vdots \\ d_{n3} \end{array} & \begin{array}{cc} \begin{array}{c} 0\\ \cdots \end{array} & \begin{array}{c} \vdots \\ 0 \end{array} \end{array} \end{array} \end{array}\right],$$ (3)

where $d_{ij}$ represents the dissimilarity between the sample points $x_{i}$ and $x_{j}$ .

DEFINITION 3 Dynamic radius r of data set X :

$$r=\frac{\mathrm{Mean}\_\mathrm{r(d)}}{T},$$ (4)

among them

$$\mathrm{Mean\_r(d)}=\frac{1}{n^{2}}\overset{n}{\underset{\mathrm{i=}1}{\sum }}\overset{n}{\underset{\mathrm{j=}1}{\sum }}d_{\mathrm{ij}},$$ (5)

T is the dynamic radius parameter, and the value is as follows:

T=−0.423+0.328K−1.211mead(d)+0.662max(d)+1.631min(d), (6)

where K represents the number of data categories; mean means the average of dissimilarity; max represents the maximum phase dissimilarity; and min represents the minimum phase dissimilarity.

DEFINITION 4 Sample dissimilarity density ρ :

$$\rho =\overset{n}{\underset{\mathrm{j=1}}{\sum }}\mathrm{\delta (}d_{\mathrm{ij}}\mathrm{-r),}$$ (7)

where $\delta \left(z\right)=\{\begin{array}{c} 1,\;\;\;\;\;\;\;\;\;z\leq 0\\ 0,\;\;\;\;\;\;others \end{array}$ , $\rho_{i}$ represents the dissimilarity density of the sample object $x_{i}$ , which is the number of points satisfied $d_{1i}<r$ .

**FIGURE 1:**
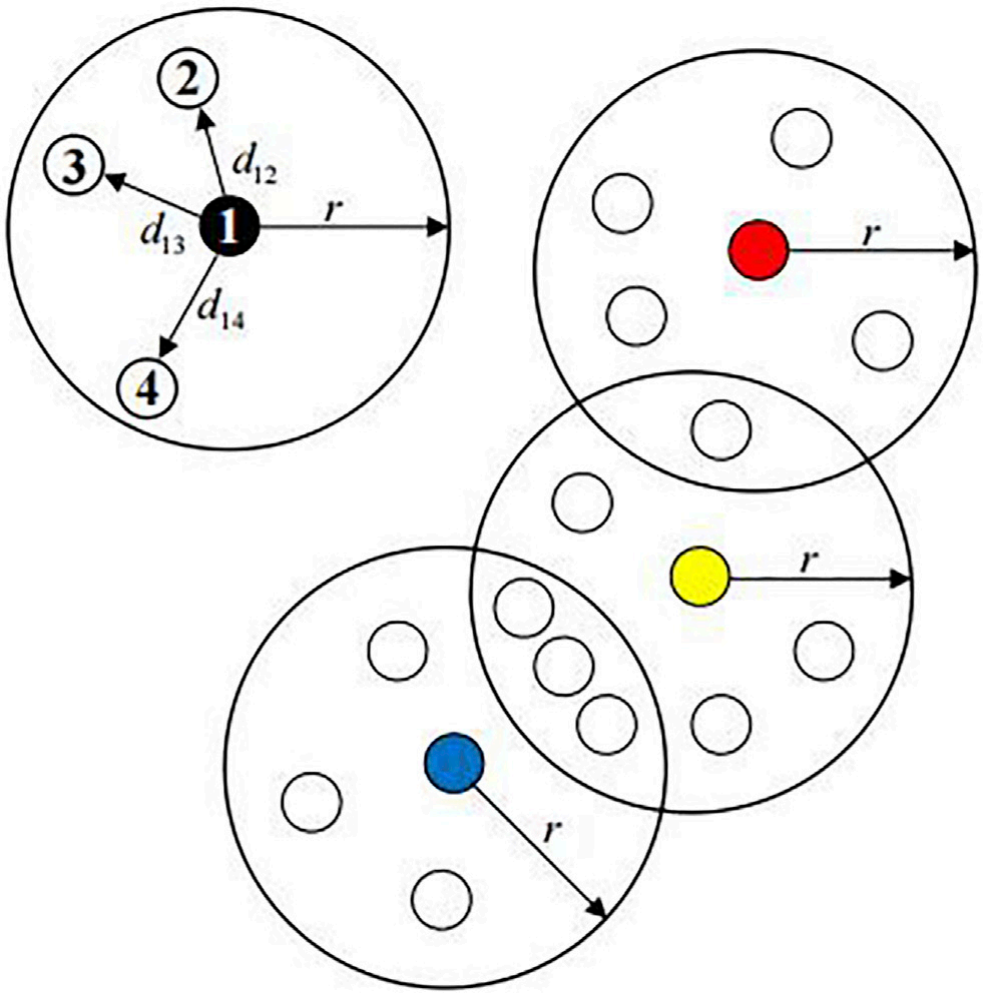
Dissimilarity density ρ .

The sample point dissimilarity density is the number of points that satisfy $d_{1i}<r$ . As shown in Figure 1, the conditions are $d_{11},d_{12},d_{13},d_{14}$ . so the dissimilarity density of sample point 1 is 4. Similarly, the dissimilarity density of red dots is 6; the dissimilarity density of yellow dots is 8; the dissimilarity density of blue dots is 7. It is to be noted that the points in the intersection of two great circles can be calculated repeatedly.

DEFINITION 5 According to Definition 4, ρ is the number of samples whose dissimilarity with the sample object $x_{i}$ is less than the dynamic radius r . Samples meeting the conditions form a candidate cluster, where the average dissimilarity between the samples of the candidate cluster is

$$\alpha \left(i\right)=\frac{1}{n^{2}}\overset{n}{\underset{i=1}{\sum }}\overset{n}{\underset{j=1}{\sum }}d_{\mathrm{ij}},$$ (8)

DEFINITION 6 The dissimilarity $s_{i}$ of candidate clusters represents the dissimilarity between sample $x_{i}$ objects $x_{j}$ , which satisfies the following formula

$$s_{i}=\{\begin{array}{cc} \min\left(d_{\mathrm{ij}}\right), & \exists p\left(j\right)\mathrm{>p}\left(i\right)\\ \max\left(d_{\mathrm{ij}}\right), & \exists p\left(j\right)\mathrm{\leq p}\left(i\right) \end{array},$$ (9)

**FIGURE 2:**
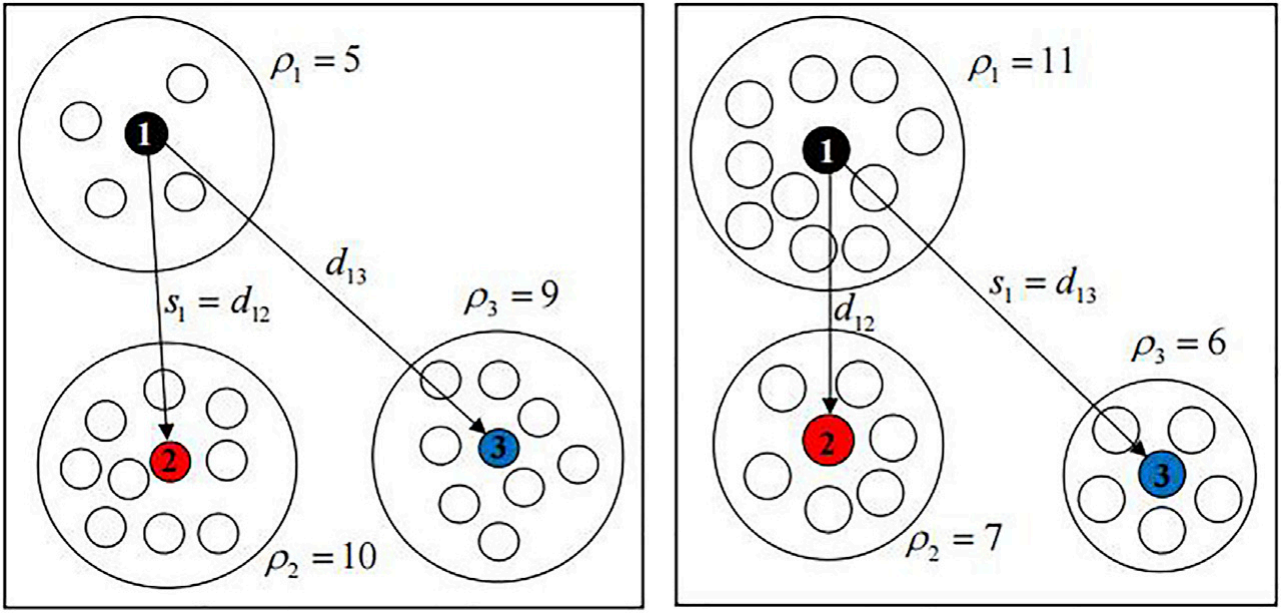
Dissimilarity of candidate clusters $s_{i}$ .

As shown on the left of Figure 2, the dissimilarity density of sample point 1 is 5, and there is a dissimilarity density larger than it, so the smallest dissimilarity is selected as the candidate cluster dissimilarity of sample point 1; as shown in the right of Figure 2, the dissimilarity density of sample point 1 is 11, and there is no dissimilarity density larger than it. Therefore, the biggest dissimilarity is selected as the candidate cluster dissimilarity of sample point 1.

By analyzing Definitions 5, 6, when the candidate cluster is formed with $x_{i}$ as the center point, if the average dissimilarity value α(i) between samples of the candidate cluster is smaller, the dissimilarity of the cluster is very small, and the similarity is very high; similarly, the greater the value of $s_{i}$ , the greater the dissimilarity between samples. Therefore, the dissimilarity density ρ , the average dissimilarity α(i) , and the dissimilarity value $s_{i}$ of candidate clusters can be taken as the standard to measure the initial center point, which is specifically defined as follows:

DEFINITION 7 The dissimilarity weight formula for selecting the cluster center point is as follows:

$$\omega_{i}=\rho_{i}*\frac{1}{\alpha_{i}}*s_{i},$$ (10)

among them, the point with the most significant weight of dissimilarity is the initial center point.

### 2.3 Algorithm Flow and Block Diagram

#### 2.3.1 Algorithm Flow

The Dissimilarity-Density-Dynamic Radius-K-means algorithm calculates the dissimilarity density ρ of sample points, the average dissimilarity α of candidate clusters, and the dissimilarity value s of candidate clusters to obtain the dissimilarity weight ω of sample points and determine a group of initial center points. Then, the obtained center point is used as the initial center point of K-means for clustering. The flow of the Dissimilarity-Density-Dynamic Radius-K-means algorithm is as follows:

1) Giving a data set $X=\left\{x_{1},x_{2},\cdots ,x_{n}\right\}$ ;
2) Calculating the dissimilarity density of all points in x is in accordance with the definition and form a set ρ ;
3) Finding that point corresponding to the maximum value from the dissimilarity set ρ ; if the number of the value is 1, the point is taken as the first initial clustering center point; if the number of the maximum value is not 1, the calculated $sum\left(i\right)=\overset{n}{\underset{j=1}{\sum }}d_{ij}$ , wherein $d_{ij}\leq r,j=1,2,\cdots ,n$ , and form the set S , that satisfying sum(i)=min(S) point is taken as the first initial center point;
4) Obtaining a first initial clustering center point at this time, recording C1 , and putting it in the set C at that time C={C1} . Then, points satisfying $d_{1i}<r$ are then removed from the data set X ;
5) Calculating the weight value $\omega_{i}$ of the dissimilarity of the remaining point according to the definition, wherein the second initial center point is the point with the maxumun weight value of the distinction and is recorded C2 and put in set C at that time C={C1,C2} . Then, deleting the points that meet the criteria;
6) Repeating step 5 until that data set is empty, C={C1,C2,⋯,Ck} ;
7) At this time, a group of initial center points C and the number K of clustering have been obtained, and the parameters are brought into the k-means algorithm for clustering;
8) Calculating the distance between each point in the sample and the initial center point, classifying the space into the cluster where the center point with the smallest distance between each other is located, and calculating the new center points of each group;
9) Repeating the step 8 until the division condition of all sample points remain unchanged or the central point does not change;
10) Output that clustering result.

#### 2.3.2 Algorithm Block Diagram

**FIGURE 3:**
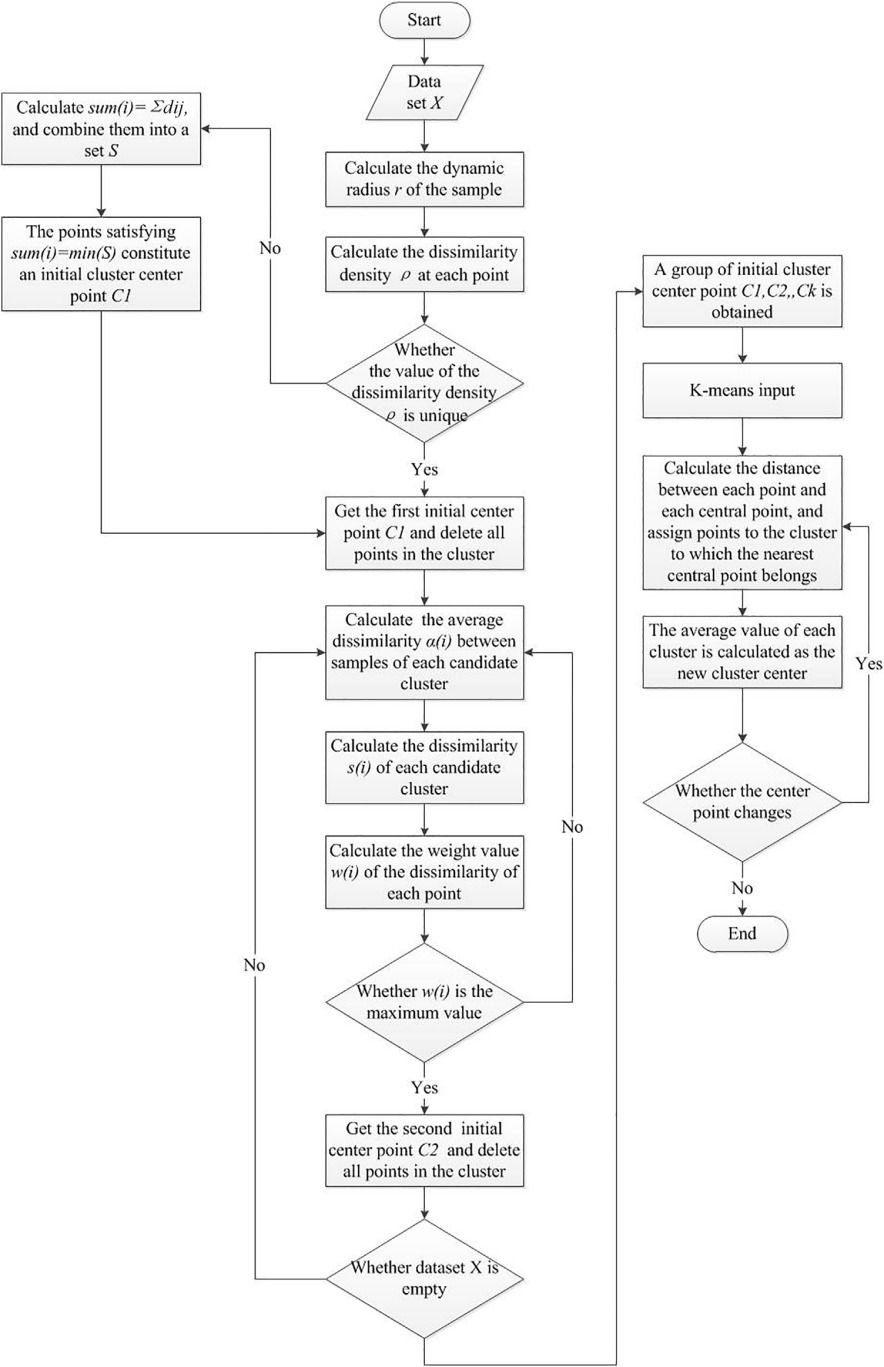
Algorithm block diagram.

The algorithm block diagram is shown in Figure 3.

### 2.4 The Necessity of Setting the Dynamic Radius Parameter T

**FIGURE 4:**
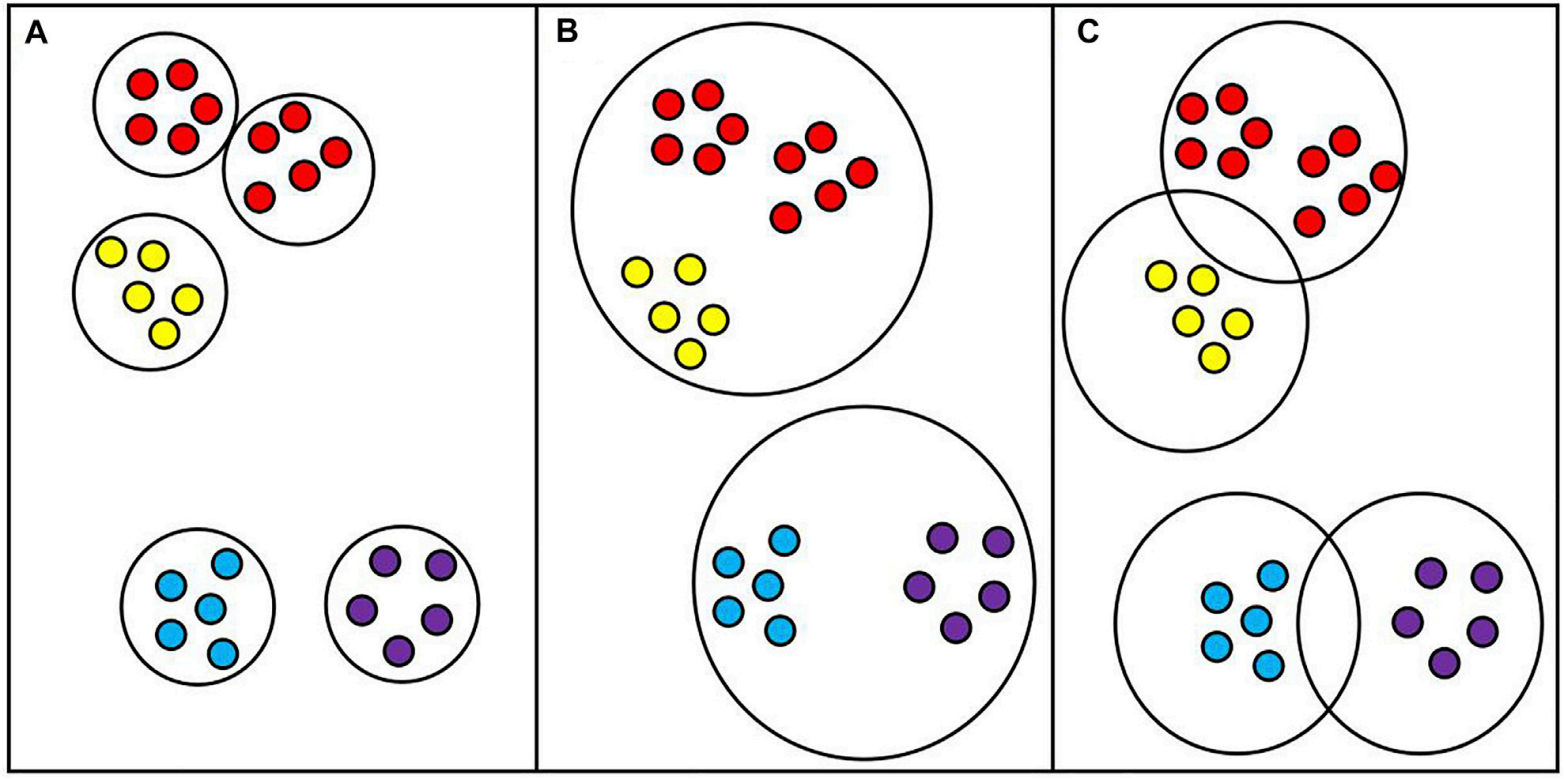
Clustering at a fixed radius. **(A)**: The radius is too small; **(B)**: The radius is too large; **(C)**: The radius is appropriate.

When introducing the D3K algorithm, we put forward the definition of dynamic radius R; the so-called dynamic radius is the ratio of average dissimilarity and dynamic radius parameter T. The distribution of the data set is not uniform. If the distribution of the data set is too scattered or too close, the average dissimilarity will be too large or too small. If the average dissimilarity is taken as the radius, the clustering result will be inaccurate, which will affect the selection of the initial center point and result in an inaccurate clustering result. If the dynamic radius parameter is added, the radius can be adjusted flexibly according to the data characteristics so as to optimize the clustering result. As shown in Figure 4 below:

As shown in Figure 4C, for clustering results under ideal conditions, appropriate radii are set and clusters are divided reasonably. However, if the average dissimilarity is taken as the radius, the average dissimilarity will be too small for some overly tight data sets, which will make the radius smaller, and the original cluster will be divided into two or more clusters, as shown in Figure 4A. For some data sets that are too scattered or have noise points, the average dissimilarity will be very large. In this case, taking the average dissimilarity as the radius will make the radius very large so that originally different clusters can be divided into one cluster, as shown in Figure 4B. Therefore, adding the dynamic radius parameter T into the model can reasonably adjust the radius size according to the data characteristics and optimize the result of cluster division.

The dynamic radius parameter T is considered from multiple perspectives, including the maximum, minimum, average, and the number of clusters K. Considering many aspects, we get the optimal solution through the greedy algorithm and then fit the equation of the dynamic radius parameter T through a large amount of data. Among them, the dissimilarity between each point and itself is 0, so the dissimilarity between each point and itself should be removed when selecting the minimum value of phase dissimilarity, that is, the value with the smallest foreign phase dissimilarity except 0. By observing the equation of dynamic radius parameter T, it is found that the coefficient of K value of the number of clusters is only 0.328, indicating that although the dynamic radius parameter T is related to the number of clusters, it does not account for the main factor, and the optimal solution of T is in an interval, so the equation can be satisfied without a particularly accurate K value.

## 3 Result

To verify the algorithm, we selected nine groups of single-cell data sets for experiments, namely, Kolod, Pollen, Ting, Ioh, Goolam, Usoskin, Xin, Zeisel, and Macosko data sets. Table 1 shows the details of the data set.

**TABLE 1:** Summary of six scRNA-seq data sets used in this study.

Data set	The number of cells	The number of genes	The number of clusters
Kolod	704	10685	3
Pollen	249	14805	11
Ting	114	11405	5
Ioh	429	18087	8
Goolam	124	16384	5
Usoskin	622	17772	4
Xin	1600	39851	8
Zeisel	3005	4412	48
Macosko	6418	12822	39

Clustering the data in Table 1 after dimension reduction can improve the clustering effect and visually analyze the clustering results. We compare the effects of six dimensionality reduction methods on single-cell data and visually examine the clustering results and find out an algorithm suitable for dimensionality reduction of single-cell data. At the same time, to verify the quality of the algorithm, we compare it with other single-cell clustering algorithms and finally confirm the selection of parameter T in this study.

### 3.1 Dimension Reduction

To find a dimension reduction algorithm suitable for single-cell data sets, we preprocess single-cell data with different dimension reduction algorithms and then cluster the reduced data to obtain clustering results. Here, we compare six dimensionality reduction algorithms: T-SNE, PCA, MDS, LPP, and LLE Isomap. By reducing dimensions in clustering, we obtain the data in Table 2:

**TABLE 2:** Clustering indexes after dimensionality reduction.

		Kolod	Pollen	Usoskin	Ting	loh	Goolam	Xin	Zeisel	Macosko
Original Data	NMI	0.5202	0.8533	0.3139	0.7262	0.5512	0.6218	0.5338	0.5262	0.4772
	FM	0.8207	0.7837	0.5923	0.534	0.6013	0.7605	0.5468	0.3260	0.3726
	Accuracy	0.6960	0.7807	0.5907	0.7746	0.5734	0.8097	0.8744	0.4985	0.4399
	RandIndex	0.7080	0.9323	0.7011	0.8370	0.7924	0.8140	0.6971	0.9230	0.9092
t-SNE	NMI	**0.8344**	**0.9169**	**0.7197**	**0.8402**	**0.8296**	**0.7298**	**0.6087**	**0.5741**	**0.6954**
	FM	**0.9025**	**0.8682**	**0.8032**	**0.9494**	**0.8540**	**0.9363**	**0.5456**	**0.3564**	**0.5339**
	Accuracy	**0.9071**	**0.9149**	**0.6521**	**0.9033**	**0.8748**	**0.8952**	**0.9306**	**0.5784**	**0.6790**
	RandIndex	**0.9005**	**0.5335**	**0.8804**	**0.9197**	**0.9459**	**0.8937**	**0.7088**	**0.9297**	**0.9488**
PCA	NMI	0.5557	0.8190	0.3435	0.8318	0.6398	0.6674	0.5821	0.4031	0.3433
	FM	0.7710	0.8013	0.5486	0.9077	0.6616	0.7779	0.5873	0.2254	0.2456
	Accuracy	0.7685	0.8233	0.5723	0.8947	0.6727	0.8653	0.9175	0.4254	0.3398
	RandIndex	0.7905	0.9475	0.6837	0.9127	0.8648	0.8679	0.7119	0.9188	0.9185
MDS	NMI	0.5519	0.8123	0.3438	0.8228	0.6444	0.7202	0.5960	0.4033	0.3441
	FM	0.7679	0.5588	0.5588	0.8429	0.6674	0.7927	0.6046	0.2255	0.2420
	Accuracy	0.7648	0.5723	0.5723	0.8596	0.6681	0.8871	0.9219	0.4252	0.3363
	RandIndex	0.7883	0.6845	0.6845	0.9067	0.8647	0.9078	0.7288	0.9189	0.9163
Isomap	NMI	0.4574	0.7350	0.3686	0.9173	0.7812	0.6535	0.6002	0.5338	0.5063
	FM	0.7797	0.6632	0.6709	0.8064	0.8292	0.7295	0.5852	0.3307	0.4182
	Accuracy	0.7741	0.6908	0.6672	0.8684	0.8436	0.8734	0.9207	0.5196	0.4634
	RandIndex	0.7590	0.9070	0.7372	0.9104	0.9355	0.8173	0.7240	0.9251	0.9133
LLE	NMI	0.5358	0.8941	0.4951	0.8172	0.7867	0.7205	0.5831	0.5719	0.6020
	FM	0.8006	0.8931	0.7353	0.8458	0.7843	0.3620	0.6042	0.3620	0.5398
	Accuracy	0.7955	0.9076	0.7267	0.8772	0.8462	0.5237	0.8882	0.5237	0.5734
	RandIndex	0.7897	0.9695	0.7841	0.8763	0.9225	0.8978	0.7240	0.8978	0.9405
LPP	NMI	0.7105	0.8875	0.6887	0.7869	0.7709	0.7056	0.5543	0.4819	0.4517
	FM	0.7977	0.8460	0.8559	0.8351	0.7449	0.7991	0.5506	0.2664	0.3275
	Accuracy	0.7979	0.8594	0.8376	0.8509	0.8089	0.8790	0.9006	0.4516	0.4020
	RandIndex	0.7925	0.9620	0.8680	0.8572	0.8693	0.8996	0.7036	0.9197	0.9232

By analyzing the data in Table 2, it can be found that after dimensionality reduction is used, the values of each index of clustering have been significantly improved, indicating that dimensionality reduction is very important for clustering, which can not only greatly increase the accuracy of clustering but also reduce the calculation time. At the same time, it can be found that in most of the data, the t-SNE algorithm has the best improvement effect. Therefore, the T-SNE algorithm can be used as an effective tool for single-cell clustering.

**FIGURE 5:**
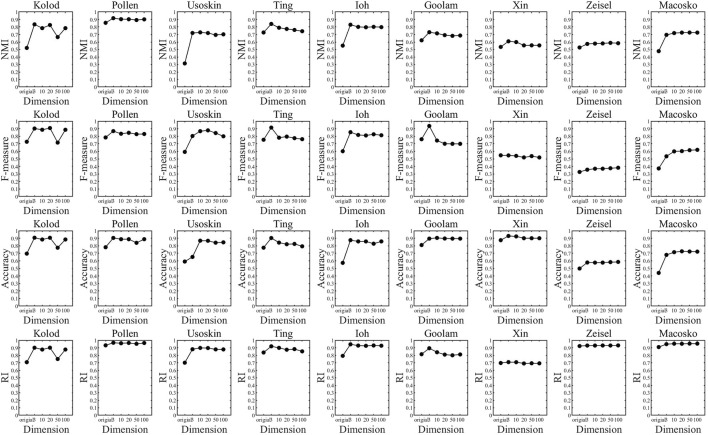
Clustering index values of different dimension clustering.

In the previous experiments, we have concluded that the t-SNE algorithm is more suitable for single-cell data dimension reduction, but how many dimensions to reduce the dimension is more suitable for clustering is still a problem to be discussed. To this end, we set up the following experiments: The t-SNE algorithm with the best dimensional reduction effect for single-cell data was selected, and six groups of single-cell data were reduced to 3, 10, 20, 50, and 100 dimensions for K-means clustering, and the clustering index results in different dimensions were analyzed. In order to compare the differences between different dimensions more clearly, the results are presented in a broken line graph. As shown in Figure 5:

Through the analysis of Figure 5, it is found that each data set has an inflection point in three dimensions, that is to say, the data will be reduced to three-dimension clusterings, and the clustering result will be significantly improved. Although some data still improve after three-dimension clustering, the increase is very small and can be almost ignored. Therefore, we can conclude that the t-SNE algorithm has the best clustering effect when the data are reduced to three dimensional ones. Therefore, in the following experiments, we uniformly used the t-SNE algorithm to reduce single-cell data to three dimensional ones for clustering.

### 3.2 Comparison With Other Clustering Algorithms

To verify the effectiveness of the D3K algorithm, we selected seven single-cell clustering algorithms to compare with it, namely, DCK (Zhang et al., 2018), S3C2 (Zhuang et al., 2021), sinNLRR (Zheng et al., 2019), Corr (Dong et al., 2018), Max-Min (Sen et al., 2018), K-means, and DBSCAN algorithm.

**FIGURE 6:**
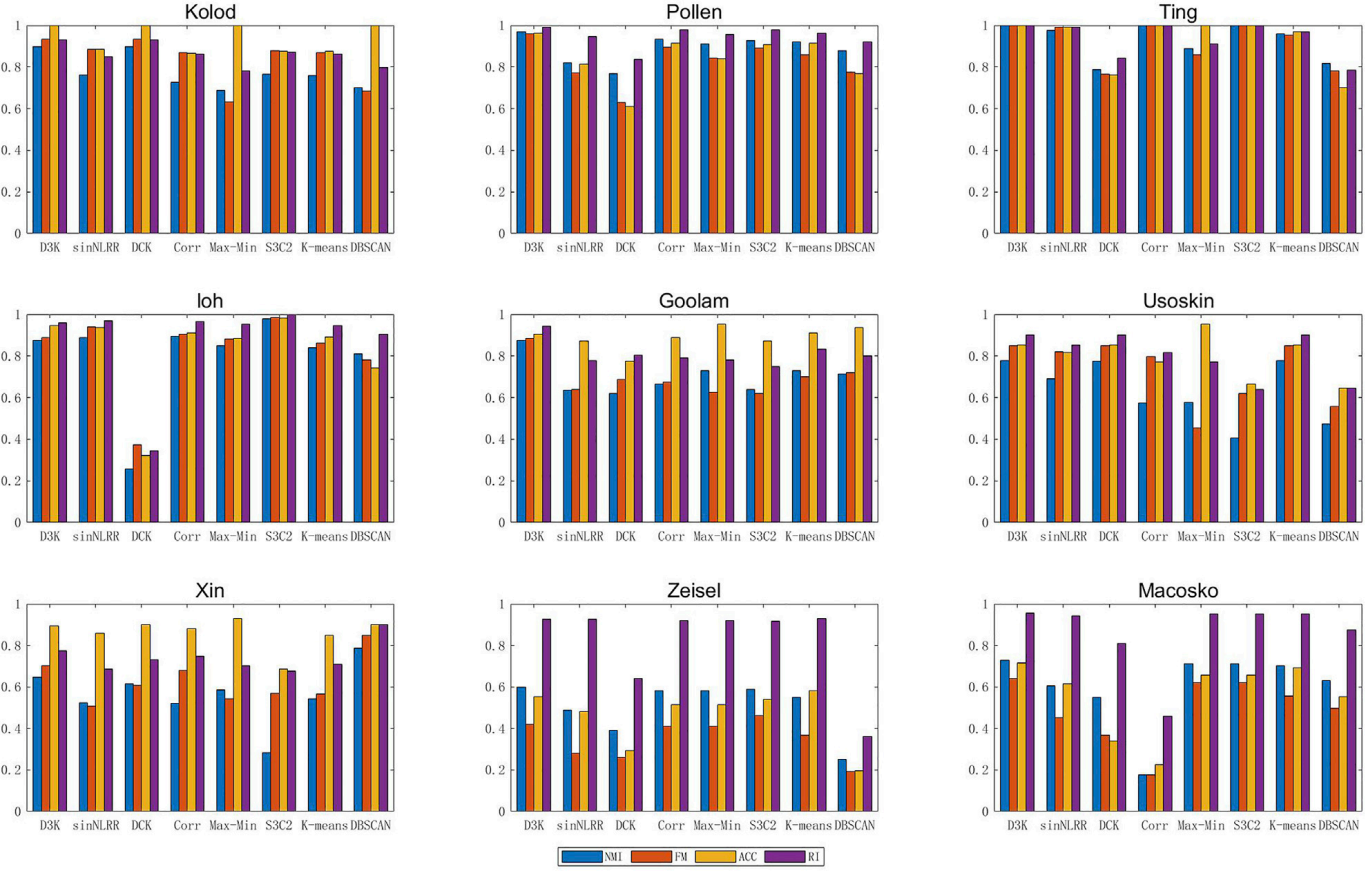
Index of the D3K algorithm in single-cell data aggregation class.

The nine groups of single-cell data in Table 1 were clustered by the single-cell clustering algorithm described above, and each index (NMI, FMeasure_node, Accuracy, RandIndex) of the clustering result was obtained to obtain Figure 6 as follows:

Compared with other clustering algorithms, the D3K algorithm is obviously higher than different algorithms in various indexes, and the results of multiple indexes are basically above 0.8, among which the effects of multiple indexes of the Pollen data set can reach above 0.95, especially Ting data set, and the results all are 1. It can be seen that the D3K algorithm can achieve ideal clustering results for both small and large data sets and can be used as a clustering model for single-cell data.

**FIGURE 7:**
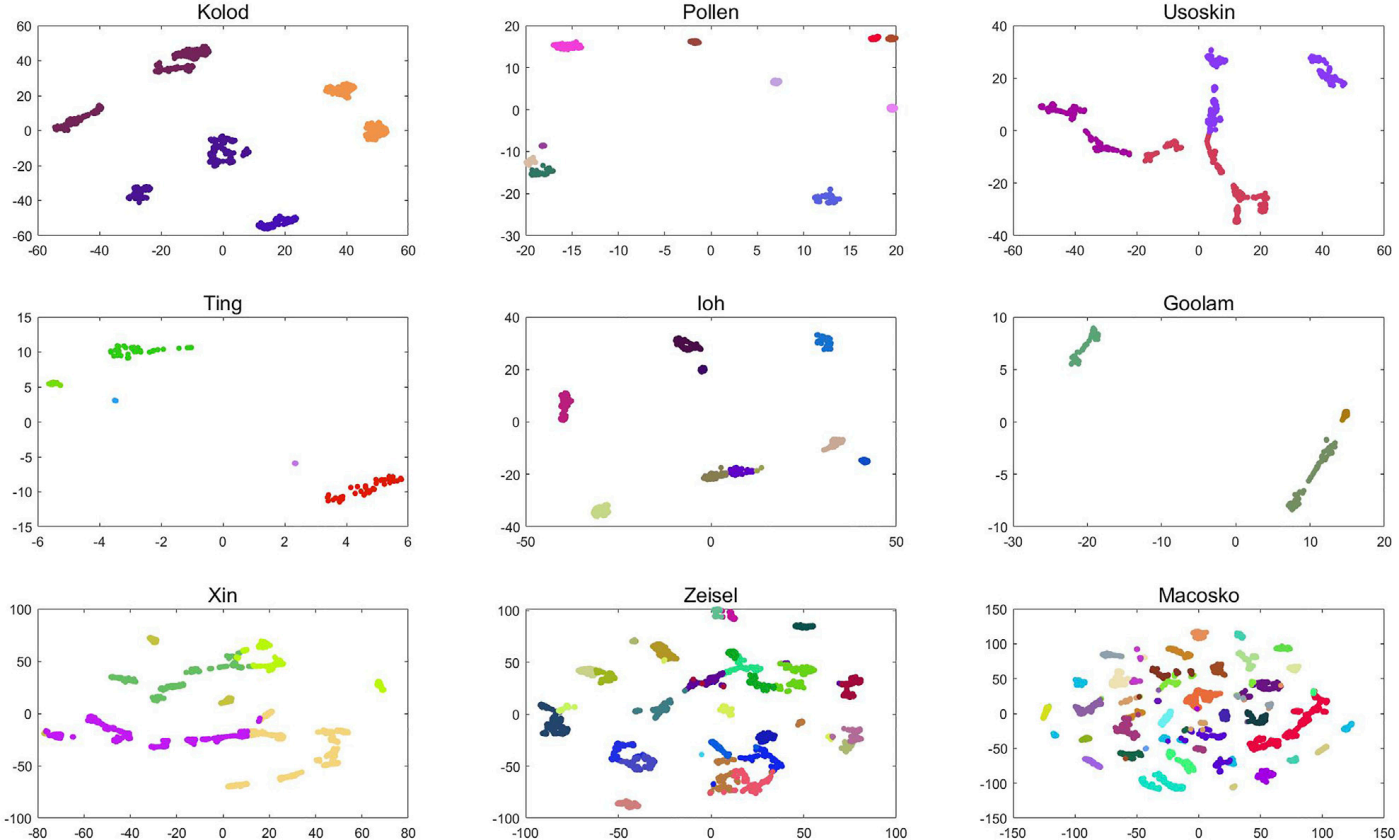
D3K algorithm visualization analysis.

Visual analysis of clustering results can not only clearly display complex data in the form of images but also intuitively observe the differences between clusters and the size of differences within clusters. For single-cell data, this study first constructs its dissimilarity degree matrix and then obtains the cluster label of single-cell data through clustering. According to the cluster label, visual analysis of the dissimilarity matrix can not only show the clustering results of single cells after clustering but also make the distance within the same cluster smaller and the distance between different clusters larger. The following Figure 7 is a visual analysis of the clustering results of six groups of single-cell data, and the clustering results of the D3K algorithm are displayed in the form of images.

As shown in Figure 7, the visualization results of the D3K algorithm after clustering 10 groups of single-cell data are shown. It can be seen that the D3K algorithm can perfectly divide these data into different cell types according to the labels after clustering and make the differences within clusters after clustering very small, but the differences between clusters are very large.

### 3.3 Validation of Parameter T

When introducing the D3K algorithm, we propose the definition of dynamic radius r , and the so-called dynamic radius is the ratio of the average degree of difference to the dynamic radius parameter. The distribution of the dataset is not uniform, and if the dataset distribution is too scattered or too tight, it will cause the average difference to be too large or too small. If the radius is based on the average degree of difference, it will affect the selection of the initial center point, resulting in inaccurate clustering results. By adding the dynamic radius parameter, you can find the right radius for each set of data to optimize clustering results.

**FIGURE 8:**
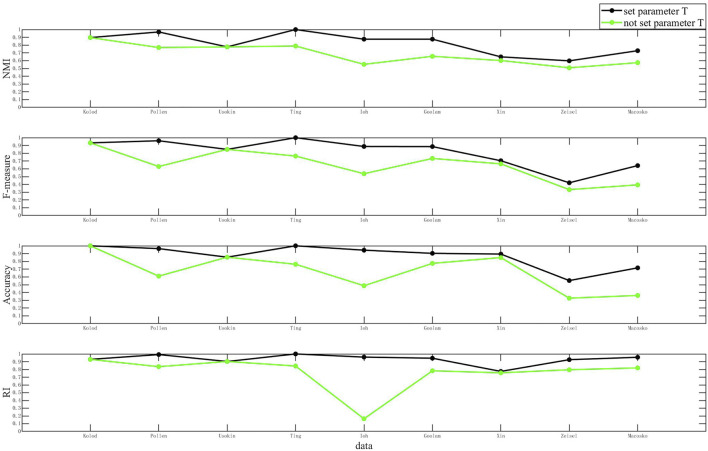
Clustering results when T and not T are set.

In order to explain the necessity of the dynamic radius parameter T more rigorously, we set up the following experiment and ninesets of single-cell data were taken and clustered using D3K. The dynamic radius parameter T is not added to the first cluster, and the dynamic radius parameter T is added to the second cluster to compare the difference between the results. The result is shown in Figure 8:

As shown in Figure 8, the comparison of clustering results of the D3K algorithm when T is set and not set is shown. The abscissa of each of these plots represents the dataset, and the ordinate coordinate represents the values of each metric. The black polyline represents the clustering result when T is set, and the green polyline represents the clustering result when T is not set. The analysis found that the clustering results when setting T were better than the clustering results when T was not set. It is to be noted that setting the T value can optimize the clustering results and make the clustering results more accurate.

### 3.4 Genetic Markers

**FIGURE 9:**
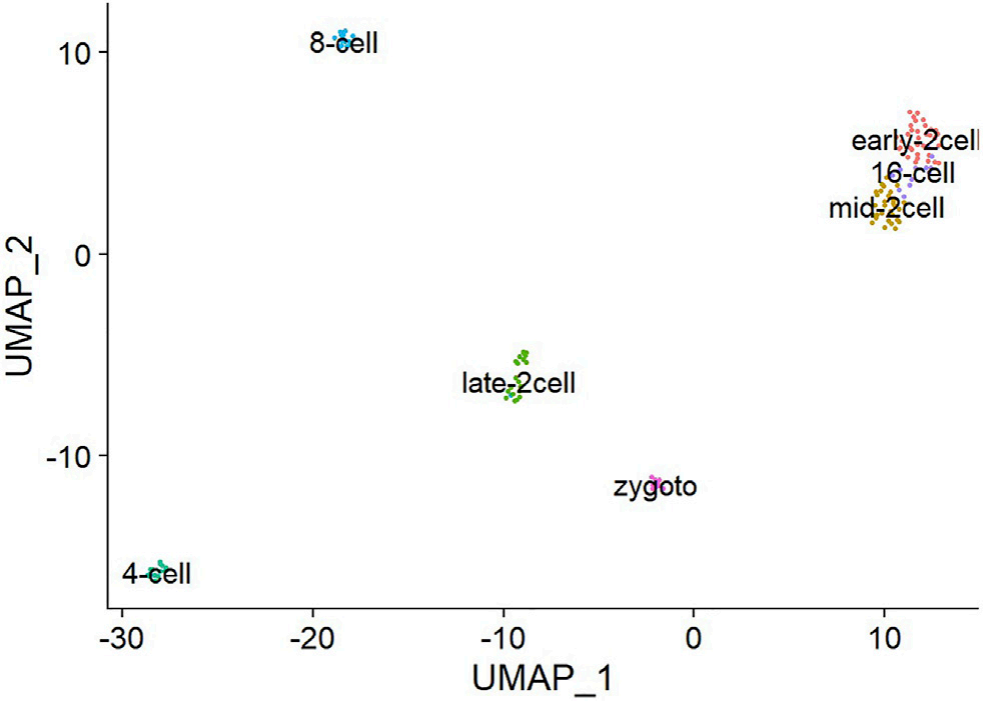
Deng data set gene marker results.

The task of single-cell scrNA-SEQ sequencing is not only to cluster single-cell sequencing data but also to cluster cells with similar gene expression patterns into the same cell type. Extraction of gene markers from the single-cell level of single-cell RNA-SEQ and cell identification is also an important part because it can assist in subsequent analysis of gene interactions. As shown in Figure 9, after annotation of the Deng data cluster class, its marker genes can be determined. The Deng marker genes include Early-2cell, mid-2cell, late-2cell, 4cell, 8cell, 16cell, and Zygoto. By clustering single-cell data, gene markers can be realized more effectively, which is convenient for further research on a single cell.

## 4 Discussion

scRNA-seq can quickly determine the precise gene expression patterns of thousands of single cells and reveal the complexity of the horizontal structure of single cells, thus improving biomedical research and solving various problems in biology. However, due to the high dimension and multi-noise characteristics of single-cell sequencing data sets, it brings significant challenges to the traditional clustering algorithm. In this study, we propose a Dissimilarity-Density-Dynamic Radius-K-means clustering algorithm. By selecting the dynamic radius, the algorithm effectively calculates the dissimilarity density ρ of the data set, the average dissimilarity α of candidate clusters, and the dissimilarity s of candidate clusters, finds a group of high-quality initial center points, and achieves the purpose of improving the K-means algorithm.

We use the Dissimilarity-Density-Dynamic Radius-K-means clustering algorithm to cluster some single-cell data sets and evaluate the clustering results. Experiments show that the Dissimilarity-Density-Dynamic Radius-K-means clustering algorithm has good performance for single-cell data clusters. At the same time, we also compared with other single-cell clustering algorithms. Experiments show that the Dissimilarity-Density-Dynamic Radius-K-means clustering algorithm is superior to other single-cell clustering algorithms.

## Data Availability

Publicly available datasets were analyzed in this study. These datasets can be found here: This study used data from the GEO database and can be found below: https://www.ncbi.nlm.nih.gov/geo/.
